# Evolving management strategies in head and neck paragangliomas: A single‐centre experience with 147 patients over a 60‐year period

**DOI:** 10.1111/coa.13380

**Published:** 2019-06-26

**Authors:** J.A. Rijken, B. de Vos, L.P. van Hest, K.M.A. Dreijerink, M. den Heijer, W. Wisselink, G.J. Blom, E.F. Hensen, C.R. Leemans

**Affiliations:** ^1^ Department of Otolaryngology/Head and Neck Surgery, Amsterdam UMC Vrije Universiteit Amsterdam Amsterdam The Netherlands; ^2^ Department of Clinical genetics, Amsterdam UMC Vrije Universiteit Amsterdam Amsterdam The Netherlands; ^3^ Department of Endocrinology, Amsterdam UMC Vrije Universiteit Amsterdam Amsterdam The Netherlands; ^4^ Department of Surgery, Amsterdam UMC Vrije Universiteit Amsterdam Amsterdam The Netherlands; ^5^ Department of Radiotherapy, Amsterdam UMC Vrije Universiteit Amsterdam Amsterdam The Netherlands; ^6^ Department of Otolaryngology/Head and Neck Surgery Leiden University Medical Center Leiden The Netherlands

**Key Points**

- Paragangliomas of the head and neck (HNPGLs) are rare, slow‐growing and usually benign tumours that arise in the paraganglion tissue associated with the autonomic nervous system.
- Management strategies and outcome of 147 patients with a total of 289 HNPGLs in a 60‐year period were evaluated.
- In HNPGLs patients, the clinician should be aware of variable clinical manifestations, such as the risk of multifocality, associated sympathetic PGL, concurrent pheochromocytoma, catecholamine excess and risk of metastatic disease.
- A decreasing number of HNPGLs were surgically resected. Increasingly, an active surveillance strategy has become the treatment of choice.
- The genetic predisposition, a key factor in the clinical risk profile (phenotype) of HNPGL patient subgroups, in combination with a better understanding of the natural course of HNPGLs, has resulted in a more conservative management of HNPGL patients.

## 1 INTRODUCTION

Paragangliomas (PGLs) are rare, slow‐growing and usually benign tumours that arise in the paraganglion tissue associated with the autonomic nervous system. PGLs can be divided into head and neck paragangliomas (HNPGLs), sympathetic paragangliomas (sPGLs) located in the abdomen or thorax, and pheochromocytomas (PHEOs) located in the adrenal glands. Generally, HNPGLs are of parasympathetic origin and about one‐third of HNPGL patients have catecholamine‐secreting tumours that may cause elevated blood pressure, palpitations, flushes and agitation.^2013^

Head and neck paragangliomas most frequently originate from the paraganglia in the bifurcation of the carotid artery, the jugular foramen, along the vagal nerve or along the tympanic nerve. Rarely, HNPGLs are located elsewhere in the head and neck region, that is, the nasal cavity, paranasal sinuses, parotid gland, cervical sympathetic chain, pharynx, larynx, trachea, aortic arch, ciliary ganglion and thyroid gland.^2011^ Most HNPGLs are characterised by slow and expansive growth, but approximately 10%‐15% of the tumours show a more aggressive, rapidly progressive behaviour.^2010^ Symptoms vary with the tumour localisation and the associated cranial nerve deficits.

Head and neck paraganglioma can occur sporadically or as part of a hereditary syndrome. PGL syndromes are mainly caused by germline mutations in genes encoding subunits or cofactors of the mitochondrial succinate dehydrogenase (*SDH*), respectively, *SDHA*, *SDHAF2*, *SDHB SDHC* and *SDHD*. An increasing number of other genes have been associated with the development of PGL, for example *RET*, *NF1*, *VHL*, *HIF2A*, *FH*, *TMEM127* or *MAX*. Different causative genes are associated with different clinical characteristics.^2014^ In the Netherlands, pathogenic variants in *SDHD* are the most prevalent cause of PGL syndrome, followed by variants in *SDHB* and *SDHA*.^2012^,^2018^ *SDHD* mutation carriers have a significant risk of developing multiple HNPGLs, with a low incidence of malignancy (1.7%). *SDHB* mutation carriers are reported to develop solitary PGLs and metastatic PGLs more frequently (7.3%).^2019^ In this study, we evaluated clinical characteristics and treatment strategies of 147 consecutive patients with a total of 289 HNPGLs referred to the department of Otolaryngology/Head and Neck surgery of the Amsterdam University Medical Centres, Vrije Universiteit Amsterdam, the Netherlands, during the last 60 years.

## 2 MATERIALS AND METHODS

Patients visiting the department between 1956 and 2017, with at least one HNPGL were included. Patient characteristics including genetic status (if available), gender, family history, age at diagnosis, number and localisation of HNPGLs, concurrent sPGL, PHEO, metastatic disease, management strategy and outcome were recorded. The duration of follow‐up was defined as the period between the date of HNPGL diagnosis (on imaging) and the most recent outpatient clinic visit. The diagnosis of HNPGL was based on patient and family history, otolaryngology examination including otoscopy and laryngoscopy, and/or computed tomography (CT) imaging, and/or magnetic resonance (MR) imaging and/or an angiography of the head and neck region including the skull base. Since 2003, HNPGL patients (and family members at risk) have been offered genetic counselling and DNA testing. Biochemical screening including the measurement of (nor)adrenaline, vanillylmandelic acid (VMA), dopamine, (nor)metanephrine and/or 3‐methoxytyramine (3‐MT) in two 24‐h urinary samples and/or plasma‐free (nor)metanephrine was offered to HNPGL patients. In case of excessive catecholamine secretion, additional radiological assessment by MR imaging or CT scans of thorax, abdomen and pelvis and/or ^123^I‐metaiodobenzylguanidine (MIBG)‐scan, and/or positron emission tomography with 2‐deoxy‐2‐[fluorine‐18] fluoro‐D‐glucose (18F‐FDG PET)‐scans/ 18F‐L‐dihydroxyphenylalanine (18F‐DOPA) PET scans were performed to identify potential sources of excessive catecholamine production outside the head and neck region. In *SDHB* mutation carriers, MR imaging of the thorax, abdomen and pelvis was performed as standard routine. Active surveillance (also called “wait and scan policy” or “watchful waiting”), radiotherapy, surgical resection or combinations were possible treatment strategies and were multidisciplinary discussed, weighing potential risks and benefits of each treatment strategy per tumour and per patient. Active surveillance, and postoperative and post‐irradiation follow‐up comprised of regular MR imaging and clinical evaluation by an endocrinologist and ENT surgeon. The interval was determined by several factors, such as tumour size, tumour progression rate, tumour localisation, symptoms and treatment modality, and thus differed per tumour and per patient. IBM SPSS Statistics version 20.0 (SPSS) was used for data analysis.

### 2.1 Ethics approval and consent to participate

The study was approved by the institutional Medical Ethics Committee (VUMC; number 2017.238). The authors declare that all procedures performed in studies involving human participants were in accordance with the ethical standards of the institutional research committee and with the 1964 Helsinki Declaration. For this type of study, formal consent is not required.

## 3 RESULTS

### 3.1 Clinical characteristics

One hundred and forty‐seven patients, 47 male (32%) and 100 female (68%), with a total of 289 HNPGLs were diagnosed in a 60‐year period. Sixty‐three patients (43%) presented with a positive family history, while the remaining 84 patients (57%) had no known family history of (HN)PGL or PHEO. The mean age at diagnosis was 45.3 years (95% CI: 42.5‐48.0) and ranged from 11 to 88 years. The mean duration of follow‐up was 13.1 years (range 0.03‐60.9, median 8.9). Four HNPGL patients (3%) developed a PHEO and two patients (1%) a sPGL. The vast majority of HNPGLs (286/289; 99%) was located at the bifurcation of the carotid artery (127/289 tumours; 44%, in 87 patients), the jugular foramen (68/289 tumours; 24%, in 63 patients), along the vagal nerve (58/289 tumours; 20% in 51 patients) or along the tympanic nerve (33/289 tumours; 11% in 32 patients). Other locations were the larynx, pharynx and nasal cavity (3/289 tumours; 1%, in three patients), and these tumours were confirmed to be PGL by histopathology. Multiple synchronous or metachronous HNPGLs were found in 79 of 147 patients (54%), up to a maximum of six metachronous HNPGL.

At diagnosis, 29 out of 96 (30%) biochemically screened HNPGL patients showed excessive catecholamine secretion. In 25 out of 29 (86%) of these patients, additional imaging was performed in order to identify the source of catecholamine excess. Two of these patients (2/29; 7%) were diagnosed with a concurrent PHEO, one of these patients was diagnosed with metastatic disease (1/29; 3%), and 2 (2/29; 7%) patients were diagnosed with a sPGL.

This percentage was 6/10 (60%) for *SDHB* patients and 16/52 (31%) for *SDHD* HNPGL patients. In three of four patients (75%) with a concurrent PHEO, excessive catecholamine secretion was present.

DNA tests were performed in 98/147 (67%) of HNPGL patients. Patient characteristics categorised per genetic subgroup are outlined in Table 1.

**Table 1:** Characteristics of 98 DNA‐tested HNPGL patients

Patient characteristics	SDHD pathogenic variant (n = 64; 65%)	SDHB pathogenic variant (n = 10; 10%)	SDHAF2 pathogenic variant (n = 1; 1%)	No SDHx pathogenic variant (n = 23; 23%)
Male/ female	20/44	4/6	0/1	7/16
Mean age at diagnosis (95% CI)	38.2 (34.9‐41.4)	45.6 (35.9‐55.3)	15	56.6 (50.7‐62.5)
Metastatic disease	3 (5%)	–	–	–
Multiple HNPGL	56 (88%)	2 (20%)	–	2 (7%)
PHEO	4 (6%)	–	–	–
sPGL	2 (3%)	–	–	–

Abbreviations: HNPGL, head and neck paraganglioma; PHEO, pheochromocytoma; sPGL, sympathetic paraganglioma.

Sixty‐four of 98 patients who had their DNA tested (65%) carried a pathogenic variant in *SDHD*, of whom 50 of 64 (78%) had a positive family history for PGL or PHEO. The p.Asp92Tyr mutation in the SDHD gene (one of the Dutch founder mutations) was the most prevalent mutation, identified in 50% of *SDHD* mutation carriers (32/64). Three of 147 HNPGL patients (2%) developed metastatic disease, defined by the occurrence of metastatic chromaffin tissue in locoregional lymph nodes or in non‐chromaffin organs distant from the primary PGL. All these three patients carried a pathogenic variant in *SDHD*. Two of three patients with metastatic disease had a concurrent PHEO. Clinical characteristics, treatment strategies and outcome of HNPGL patients with metastatic disease are outlined in Table 2. Treatment strategies and outcome for patients with a solitary HNPGL are outlined in Table 3. As different treatment strategies may apply different tumour locations within one patient, a single treatment strategy could not be associated with a patient with multiple HNPGLs.

**Table 2:** Clinical characteristics, treatment strategies and outcome of head and neck paraganglioma patients with metastatic disease

Case	Sex	Mutation	Location HNPGL	Age^a^	Age^b^	Location metastases (age)	PHEO	Treatment HNPGL (age)	Treatment malignant disease (age)	Outcome
1	F	SDHD p.Asp92Tyr	CBPL JFPR VPR	34	37	Paravertebral T7 (37) Mediastinal (47) Pulmonary (47) Cardial (53)	Yes	surgery VPR, JFPR (35) RT CBPL (37) RT VPR, JFPR (37)	RT paravertebral T7 (37)	Alive at age 53, with disease
2	M	SDHD p.Asp92Tyr	JFPR	39	47	Mediastinal	Yes	No	No	Alive at age 48, with disease
3	F	SDHD p.Asp92Tyr	CBPR CBPL	45	64	Bone (vertebra)	No	surgery CBPL, CBPR (45)	anterior corporectomy C3‐5 and partially C6 (64) Lu‐177‐octreotide therapy (65) Lu‐177‐octreotide therapy (72)	Alive at age 75, with disease

Abbreviations: CBPL, carotid body paraganglioma left; CBPR, carotid body paraganglioma right; F, female; HNPGL, head and neck paraganglioma; JFPR, jugular foramen paraganglioma right; M, male; RT, radiotherapy; VPR, vagal paraganglioma right.

a age in years at diagnosis of head and neck paraganglioma.

b age in years at diagnosis of metastatic disease.

**Table 3:** Overall treatment strategy and outcome in patients with a solitary head and neck paraganglioma

Tumour localisation	Overall outcome (%)	Mean follow‐up (y)	Treatment strategy	Treatment strategy n (%)	Tumour classification^a^							Mean follow‐up (y)	Outcome		
													NED	AWD	DID
					Shamblin										
					Type 1		Type 2		Type 3		Unknown^b^				
Carotid body tumour (n = 17)	NED 8 (47%)	11.2	Active surveillance	8 (47%)	–		5		3		–	7.7	–	8 (100%)	–
	AWD 9 (39%)		Surgery	9 (53%)	2		4		2		1	14.3	8 (89%)	1 (11%)	
	DOD 0		Radiotherapy	–	–		–		–		–	–	–	–	–
	DID 0		Surgery + adjuvant radiotherapy	–	–		–		–		–	–	–	–	–
					Fisch										
					Type C1	Type C2	Type De1	Type De 2	Type Di 1	Type Di 2	Unknown^b^				
Jugular body tumour (n = 25)	NED 3 (12%)	8.7	Active surveillance	12 (48%)	10	–	–	–	–	1	1	6.3	–	11 (92%)	1 (8%)
	AWD 19 (76%)		Surgery	3 (32%)	–	–	1	–	–	–	7	9.2	2 (25%)	4 (50%)	2 (25%)
	DOD 0		Radiotherapy	3 (12%)	2	1	–	–	–	–	–	9.8	–	3 (100%)	–
	DID 3 (12%)		Surgery + adjuvant radiotherapy	2 (8%)	–	1	–	–	1	–	–	19.9	–	2 (100%)	–
Vagal body tumour (n = 10)	NED 2 (20%)	7.2	Active surveillance	5 (50%)								2.2	–	5 (100%)	–
	AWD 8 (80%)		Surgery	4 (40%)								14.2	2 (50%)	2 (50%)	–
	DOD 0		Radiotherapy	1 (10%)								4.6	–	1 (100%)	–
	DID 0		Surgery + adjuvant radiotherapy	–								–	–	–	–
					Fisch										
					Type A			Type B			Unknown^b^				
Tympanic body tumour (n = 15)	NED 10 (66%)	3.1	Active surveillance	5 (33%)	2			3			–	1.1	–	5 (100%)	–
	AWD 5 (33%)		Surgery	10 (67%)	3			5			2	4.1	10 (100%)	–	–
	DOD 0		Radiotherapy	–	–			–			–	–	–	–	–
	DID 0		Surgery + adjuvant radiotherapy	–	–			–			–	–	–	–	–

Abbreviations: AWD, alive with disease; DID, dead due to intercurrent disease; DOD, dead of disease; NED, no evidence of disease.

a Carotid body tumour: according to Shamblin; jugular body and tympanic body tumour: according to Fisch.

b Imaging studies not available.

### 3.2 Management

**Figure 1:**
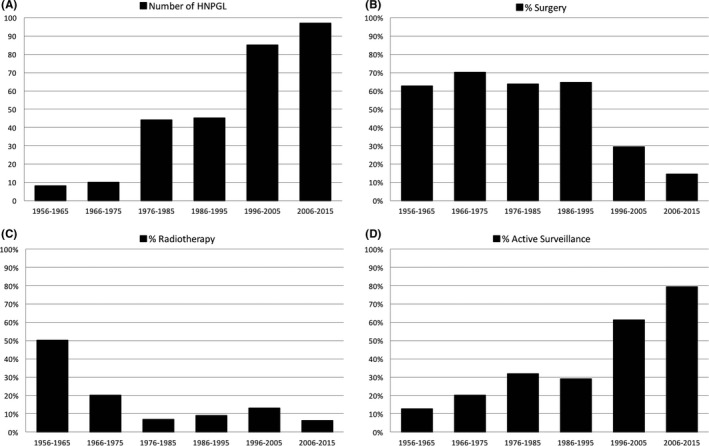
Management of head and neck paragangliomas. A, Number of diagnosed head and neck paragangliomas (HNPGLs). B, Percentage of HNPGLs that was surgically resected. C, Percentage of HNPGLs that was treated with radiotherapy. D, Percentage of HNPGL followed an active surveillance policy

Since 1956, an increasing number of HNPGLs have been diagnosed (Figure 1).

Whereas the majority (64%) of HNPGLs were surgically resected in the period 1956‐1995, in the last two decades surgery has been performed in a decreasing percentage of tumours (21%). Surgery was relatively frequently performed on solitary carotid body tumours (41%) and tympanic tumours (67%), whereas PGLs along the vagal nerve or at the jugular foramen were treated surgically in only 22% and 31%, respectively.

In the period 1956‐1965, up to 50% of HNPGLs were treated with radiotherapy. This percentage has decreased (9% in 1966‐2015) and has remained stable in the last decades. An increasing number of patients are observed (active surveillance), especially since the year 2000, coinciding with the increasing insight into the genetic determinants of PGL syndrome.

## 4 DISCUSSION

This single‐centre study describes clinical characteristics and outcome of treatment in a population HNPGL patients. In accordance with earlier reports, the vast majority of HNPGLs is located at the bifurcation of the carotid artery (59%), the jugular foramen (43%), along the vagal nerve (34%) or along the tympanic nerve (22%).^2011^ Importantly, 75/98 (77%) HNPGL patients who had their DNA tested were found to have a hereditary form of PGL. The majority of germline mutations in this single‐centre study are found in *SDHD* (65%), comparable with previous reports on HNPGL cohorts in the Netherlands.^2012^

Multifocal HNPGLs were found in 54% of the patients. Multifocality was especially prevalent in *SDHD*‐linked HNPGL patients (88%). This may have important ramifications for treatment decisions in this patient subgroup, even in apparently solitary tumours. As multifocal and bilateral tumours may occur synchronous or metachronous, bilateral cranial nerve involvement resulting in significant impairment of speech, swallowing and breathing has to be anticipated. If cranial nerve deficit occurs, it is usually better tolerated if the onset is slowly progressive, due to tumour progression, as opposed to a sudden paralysis due to surgery. In our series, 3/147 patients (2%) developed metastatic disease. Interestingly, these three patients were *SDHD* mutation carriers (3/64, 4.7%). This percentage for *SDHD* mutation carriers is in accordance with a previously published meta‐analysis.^2012^ None of the 10 *SHDB* mutation carriers proved to have metastatic disease or developed a PHEO or sPGL, an observation that is most likely due to the limited number of *SDHB*‐linked patients is in this cohort.

The last decades a rapidly expanding number of HNPGLs has been diagnosed in our centre. This increase is probably the result of intensified screening protocols and the introduction of DNA testing of HNPGL patients and cascade screening resulting in an early diagnosis of HNPGL in family members at risk. The management of HNPGL patients is topic of debate and has evolved considerably during the last decades. There is no universal best treatment option rather the optimal strategy is determined by a dedicated multidisciplinary team based on patient characteristics (such as age, condition and preferences), tumour characteristics (such as localisation, size, multifocality and associated cranial nerve deficits). Whether or not a patient has a germline pathogenic variant has become increasingly important in the clinical decision‐making, as it has become more and more clear that the genetic predisposition is a key factor in the clinical risk profile (phenotype) of HNPGL patient subgroups. Important characteristics such as the risk of multifocality, associated sPGL en PHEO, risk of metastatic disease and even mortality seem to be highly associated with the causative gene.^2019^

A surgical approach is still the treatment option of choice in the majority of carotid body and tympanic tumours, tumours that can generally be surgically resected with limited surgical risk. Growing insight into the usually indolent natural course of HNPGL has resulted in a more conservative approach of tumours in which surgery would infer considerable risk to cranial nerves, that is, vagal and jugular PGL (Figure 1). This approach has been supported by several cohort studies, describing stable or slowly progressive tumours in a large proportion of HNPGL patients (42%‐79%).^2012^,^2015^ In the Netherlands, therapeutic options (ie surgical resection, radiotherapy or surveillance) are multidisciplinary discussed, weighing potential risks and benefits of each treatment strategy per tumour and per patient.

Moving forward, more research is necessary to accurately predict the clinical behaviour of specific HNPGL tumours of individual patients, allowing for even more tailor‐made management strategies, not only with regard to the natural course of the disease, but also with regard to the short‐ and long‐term effects of possible interventions. As tumour eradication is not always possible or necessary, quality of life should be the dominant outcome parameter.

## CONFLICT OF INTEREST

The authors declare no conflict of interest.
